# Relating categorization to set summary statistics perception

**DOI:** 10.3758/s13414-019-01792-7

**Published:** 2019-06-26

**Authors:** Noam Khayat, Shaul Hochstein

**Affiliations:** Life Sciences Institute and Edmond and Lily Safra Center (ELSC) for Brain Research, Hebrew University, 91904 Jerusalem, Israel

**Keywords:** Categorization, Prototype, Boundary, Summary statistics, Ensemble, Mean, Range

## Abstract

Two cognitive processes have been explored that compensate for the limited information that can be perceived and remembered at any given moment. The first parsimonious cognitive process is object categorization. We naturally relate objects to their category, assume they share relevant category properties, often disregarding irrelevant characteristics. Another scene organizing mechanism is representing aspects of the visual world in terms of summary statistics. Spreading attention over a group of objects with some similarity, one perceives an ensemble representation of the group. Without encoding detailed information of individuals, observers process summary data concerning the group, including set mean for various features (from circle size to face expression). Just as categorization may include/depend on prototype and intercategory boundaries, so set perception includes property mean and range. We now explore common features of these processes. We previously investigated summary perception of low-level features with a rapid serial visual presentation (RSVP) paradigm and found that participants perceive both the mean and range extremes of stimulus sets, automatically, implicitly, and on-the-fly, for each RSVP sequence, independently. We now use the same experimental paradigm to test category representation of high-level objects. We find participants perceive categorical characteristics better than they code individual elements. We relate category prototype to set mean and same/different category to in/out-of-range elements, defining a direct parallel between low-level set perception and high-level categorization. The implicit effects of mean or prototype and set or category boundaries are very similar. We suggest that object categorization may share perceptual-computational mechanisms with set summary statistics perception.

Categorization is one of the most important mechanisms for facilitating perception and cognition, helping to overcome cognitive-perceptual bottlenecks (Cowan, 2001; Luck & Vogel, 1997) and perceive the “gist” of the scene (Alvarez & Oliva, 2009; Cohen, Dennet & Kanwisher, 2016; Hochstein & Ahissar, 2002; Hock, Gordon, & Whitehurst, 1974; Iordan, Greene, Beck, & Fei-Fei, 2015, 2016; Jackson-Nielsen, Cohen & Pitts, 2017; Oliva & Torralba, 2006; Posner & Keele, 1970). Categorization follows and expands on the natural categories of objects in our environment, the intrinsic correlational structure of the world (Goldstone & Hendrickson, 2010; Rosch, Mervis, Gray, Johnson, & Boyes-Braem, 1976). There is long-term debate concerning the mechanisms and cerebral sites of categorization, with recent studies suggesting that there are multiple sites and processes of categorization (Ashby & Valentin, 2017; Nosofsky, Sanders, Gerdom, Douglas, & McDaniel, 2017). Thus, categorization itself may be categorized by task or goal (Ashby & Maddox, 2011), neural circuit (Iordan et al., 2015; Nomura & Reber, 2008), utility (J. D. Smith, 2014), and context (Barsalou, 1987; Koriat & Sorka, 2015, 2017; Roth & Shoben, 1983). The most common and accepted theoretical mechanisms for categorization are still rule based, defining clear boundaries between categories (Davis & Love, 2010; Goldstone & Kersten, 2003; Sloutsky, 2003; E. E. Smith, Langston, & Nisbett, 1992) and their cortical representations (Iordan et al., 2015, 2016; Kriegeskorte et al., 2008), and prototype-based or exemplar-based, defining family resemblance (Ashby & Maddox, 2011; Goldstone & Kersten, 2003; Iordan et al., 2016; Maddox & Ashby, 1993; Medin, Altom, & Murphy, 1984; Nosofsky, 2011; Posner and Keele, 1968; Rosch, 1973; Rosch, Mervis, et al., 1976; see also Clapper, 2017).

In parallel, recent interest has focused on the perception of summary statistics of sets of stimulus elements. Observers have a reliable representation of the mean and range of sets of stimuli, even without reliable perception of the individual members of the presented set. Summary statistics, rapidly extracted from sets of similar items, presented spatially (Alvarez & Oliva, 2009; Ariely, 2001) or temporally (Corbett & Oriet, 2011; Gorea, Belkoura, & Solomon, 2014; Hubert-Wallander & Boynton, 2015), include average, and range or variance of their size (Allik, Toom, Raidvee, Averin, & Kreegipuu, 2014; Ariely, 2001; Corbett & Oriet, 2011; Morgan, Chubb, & Solomon, 2008; Solomon, 2010), orientation (Alvarez & Oliva, 2009), brightness (Bauer, 2009), spatial position (Alvarez & Oliva, 2008), and speed and direction of motion (Sweeny, Haroz, & Whitney, 2013). Extraction of summary statistics appears to be a general mechanism operating on various stimulus attributes, including low-level information, as mentioned above, and more complex characteristics, such as facial expression (emotion) and gender (Haberman & Whitney, 2007, 2009; Neumann, Schweinberger, & Burton, 2013), object lifelikeness (Yamanashi-Leib, Kosovicheva, & Whitney, 2016), biological motion of human crowds (Sweeny, Haroz, & Whitney, 2013), and even numerical averaging (Brezis, Bronfman, & Usher, 2015; for recent reviews, see Bauer, 2015; Cohen et al., 2016; Haberman & Whitney, 2012; Hochstein, Pavlovskaya, Bonneh, & Soroker, 2015). Examples of the methods used in these studies are shown in Fig. 1; see methodological details in the figure caption.

**Fig. 1:**
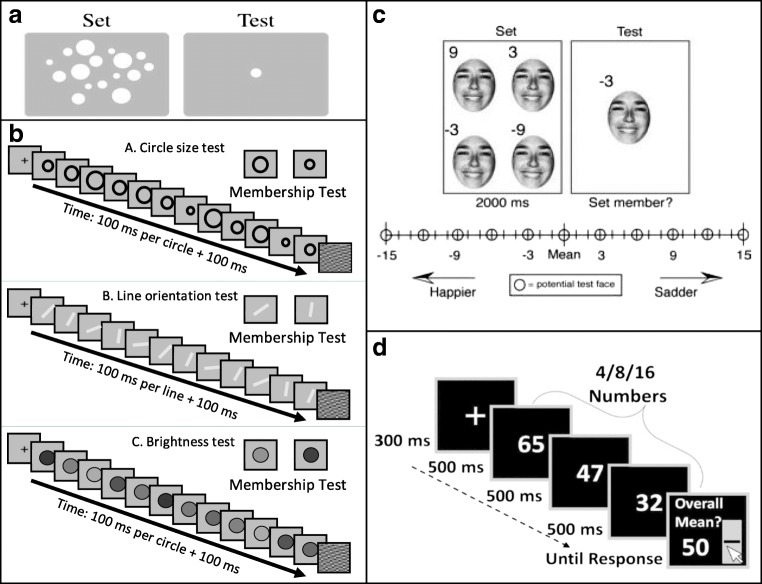
Previous study stimulus sets. a Ariely’s (2001) schematic representation of the two intervals used in his experiment’s trials. Observers were exposed for 500 ms to a set of spatially dispersed circles differing by size and then asked if a test stimulus size had been present in the set, or, is smaller/larger than the set mean. **b** Khayat and Hochstein’s (2018) RSVP sequences consisted of 12 elements, each presented for 100 ms plus 100 ms interstimulus interval (ISI), followed by a two-alternative forced-choice (2-AFC) membership test (i.e., which test element had been present in the sequence). Blocks contained circles differing in size, lines differing in orientation, or discs differing in brightness. Observers were asked which of two test elements was present in the set. They were unaware that either test element could equal the set mean or the nonmember could be outside the set range. **c** Haberman and Whitney’s (2009) task included four faces (from a set of 4, 8, 12, or 16), differing in facial emotional expression, presented for 2 s. Observers then indicated whether the test face was a member of the set, or was happier/sadder than the set mean. **d** Brezis et al.’s (2015) trials consisted of two-digit numbers sequentially presented in a rate of 500 ms/stimulus. Set size was 4, 8, or 16. Participants reported their estimate of the set average

We have suggested that these phenomena, categorization and set perception, may be related since they share basic characteristics (Hochstein 2016a, 2016b; Hochstein, Khayat, Pavlovskaya, Bonneh, & Soroker, 2018). In both cases, when viewing somewhat similar, but certainly not identical items, we consider them as if they were the same, as a shortcut to representing them and prescribing a single appropriate response (Ariely, 2001; Medin, 1989; Rosch & Mervis, 1975; Rosch, Mervis, et al., 1976). When we globally spread attention, and see a flock of sheep in a meadow, a shelf of alcohol bottles at a bar, a line of cars in traffic, or a copse of trees in a forest, we are both categorizing these objects as sheep, alcohol bottles, cars, and trees, and relating to the average properties of each set. Similarly, in laboratory experiments, we present a set of circles (Alvarez & Oliva, 2008; Ariely, 2001; Corbett & Oriet, 2011; Khayat & Hochstein, 2018), line segments (Khayat & Hochstein, 2018; Robitaille & Harris, 2011), or faces (Haberman & Whitney, 2007, 2009), and observers perceive the nature of the images as circles, lines, or faces and relate to their average properties. All animals in the category “dogs” have four legs and a tail, but they may vary in color, size, and so forth. All circles in a set are round, though they may vary in size or brightness. Categorization emphasizes relevant or common properties and deemphasizes irrelevant or uncommon properties, reducing differences among category members (Fabre-Thorpe, 2011; Goldstone & Hendrickson, 2010; Hammer, Diesendruck, Weinshall, & Hochstein, 2009; Rosch, Mervis, et al., 1976; Rosch, Mervis, et al., 1999, Rosch & Lloyd, 1978, Rosch 2002). Similarly, set perception captures summary statistics without noting individual values. Categorization, like ensemble perception, may depend on rapid feature extraction, to determine presence of defining characteristics of objects.

In particular, set perception includes set mean and range (Ariely, 2001; Chong & Treisman, 2003, 2005; Khayat & Hochstein, 2018; Hochstein et al., 2018), and categorization might rely on the related properties of prototype (or mean exemplar; e.g. Ashby & Maddox, 2011) and/or intercategory boundaries (or category range; e.g., Goldstone & Kersten, 2003). This conceptual similarity has been confirmed by the recent finding that set characteristics are perceived implicitly and automatically (Khayat & Hochstein, 2018), just as objects are categorized implicitly and automatically at their basic category level (Potter & Hagmann, 2015; Rosch, Mervis, et al., 1976). Finally, it has been suggested that determining whether a group of objects in a scene belong to the same category may actually depend on their characteristics that allow them to be seen as a set (Utochkin, 2015). The similarities of categories and sets led us to ask if the detailed properties of their perception are also similar, so that it may be hypothesized that similar mechanisms are responsible for their cerebral representation.

The goal of the current research is to detail the similarity between set and category perception by applying to categories the very same tests that we used to study implicit set perception (Khayat & Hochstein, 2018). The following section briefly reviews the results of these previous tests.

We note in advance that there are important differences between categorization and set perception. Object categories are learned over a lifetime of experience, while set ensemble statistics can be acquired on the fly. Different life experience may lead to individual differences in categorization and choice of object seen as the category prototype. Categorization may involve semantic processes, while set perception has been demonstrated for simple visual features (though including face emotion). Thus, it would be difficult to claim that ensemble perception and categorization are identical, or take place at the same cortical site. However, their being different makes comparing them even more important, since if they share essential properties, they may depend on similar or analogous processes, albeit at different cortical sites. This is the aim of the current study.

## Previous study

We studied implicit perception and memory of set statistics by presenting a rapid serial visual presentation (RSVP) sequence of images of items differing by low-level properties (circles of different size, lines of different orientation, discs of different brightness; see Fig. 1b), and testing only memory of the seen members of the sequence (Khayat & Hochstein, 2018). Note that the mean of the set—the mean size circle, mean orientation line, or mean brightness disk—was sometimes included in the set sequence and sometimes not. Following set RSVP presentation, we presented two images simultaneously, side by side. One of these images was of an item that had been seen in the image sequence—the SEEN item—and one was a NEW item, not seen in the sequence. Observer memory was tested by asking participants to choose which of the two simultaneously presented image items had been seen in the sequence. Participants were informed that always one item had been SEEN and one would be NEW. We did not inform them that sometimes one test element would have the property that was the mean of all of the items presented in the sequence and that this test item could be the SEEN item (i.e., a member of the RSVP sequence, in which case it was, of course, included in the sequence) or it could be the NEW item, (i.e., not a member of the previously viewed sequence, and thus, in this case, it had not been presented in the RSVP sequence). We also did not inform them that sometimes the NEW, non-sequence-member was outside the range of the properties of the seen sequence elements. Not mentioning to the participants the words “mean” and “range,” the goal was to test whether observers would automatically perceive set mean property and choose the test item that matched this mean—irrespective of whether this test item was the one that had been seen in the sequence or if it was the foil, the test item that was new and never been seen before. Similarly, would observers automatically perceive the range of the properties of the set and easily reject foils that were outside the range of the items in the sequence?

We call these test-stimulus contingencies trial subtypes, as shown in Table 1. We indicate as “in” test elements within the range of the variable property of the sequence; “out” indicates an element with this property outside that range, and “mean” indicates a test element with test property equal to the mean of all those in the sequence (note that to be the mean, the element must be “in” the sequence property range). Test stimuli consist of a pair of images, one SEEN and one NEW, and we indicate the pair with two mnemonics: the first mnemonic refers to the test element SEEN in the sequence; the second to the NEW, never-before-seen element, as follows: SEENmean–NEWin (test element that was SEEN in the sequence equals the set mean; both elements in the range of the variable property in the sequence); SEENin–NEWmean (the property of the never-seen NEW test element equals the mean of the seen sequence elements); SEENin–NEWin (both test elements have the property within the range of the sequence elements, but neither equals their mean); SEENmean-NEWout and SEENin-NEWout (the property of the NEW, never-seen element is outside the set range, and the property of the SEEN test element is either equal to the mean or just in the sequence range).

**Table 1:** Member recall test trial subtypes

SEEN test image (correct)	NEW test image (Incorrect)	Expected performance
SEENmean	NEWout	Best
SEENin	NEWout	Better
SEENmean	NEWin	Better
SEENin	NEWin	Baseline
SEENin	NEWmean	Worse

*Note.* Test image elements could be both from the RSVP sequence (“in”), one could be the mean (“mean”) of that sequence (whether presented, SEENmean, or not, NEWmean), and the NEW element image could be out of the sequence range (NEWout). On every trial, one element image had been SEEN in the sequence, and the other was not (i.e., NEW). Test pairs of the baseline subtype have both SEEN and NEW objects from the sequence range, one actually present and one not, and neither is the mean. If participants have difficulty recalling all elements in the sequence, but perceive and recall the mean of the sequence, we expect better performance when the SEEN test element equals the mean, and worse performance when the NEW element equals the mean. If participants perceive the range of the sequence elements, we expect better performance when the NEW element is outside the range and easily rejected. Trial subtypes were presented in randomized order, without observers knowing about this classification

**Fig. 2:**
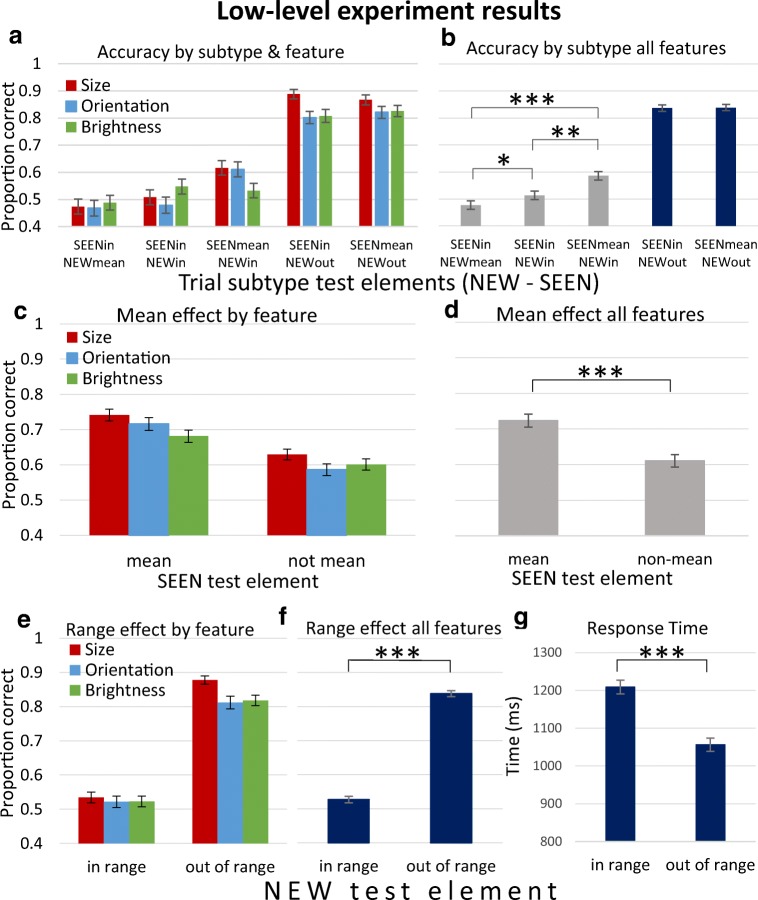
Low-level experiment results. **a** Accuracy rates for each trial subtype (i.e., their test elements); SEEN versus NEW being equal to the set sequence mean (“mean”), being in the set range (“in”) or outside the range (“out”), and each stimulus feature (colored bars; see legend). Thus, trial subtypes include: SEENmean–NEWin (seen test element = mean; both test elements in sequence range); SEENin–NEWmean (new test element = mean; both in sequence range); SEENin–NEWin (neither = mean; both in sequence range); SEENmean–NEWout (seen test element = mean; new element outside sequence range); SEENin–NEWout (seen test element not = mean; new test element outside sequence range). **b** Accuracy rates for each trial subtype, averaged across stimulus features. **c** Mean effect for each stimulus feature; accuracy rates for trials where the SEEN test element equaled the set mean versus when it differed from the mean. Each comparison is significant, *p* < .05. **d** Mean effect across features, *p* < .001. **e** Range effect for each stimulus feature; accuracy rates for trials where the NEW test element is in range versus out of range. Each comparison is significant, *p* < .01. **f** Range effect across features, *p* < .001. **g** Range effect seen in response time, indicating this is not an accuracy–time trade-off, *p* < .001. All results from Khayat and Hochstein (2018). Error bars here and in all following graphs represent between-participant standard error of the mean. (Color figure online)

As demonstrated in Fig. 2a–d, we found a mean effect for each of the three variables tested, circle size, line orientation, and disk brightness: Participants chose the test element with the property that was equal to the mean more often, whether it was the SEEN element (SEENmean–NEWin), or the NEW element (SEENin–NEWmean), compared with the case where both were in the sequence range, but neither was the mean (SEENin–NEWin).

We concluded that since the stimulus sequence was quite rapid, participants had difficulty remembering all the members of the RSVP set, and maybe even any one of them. Instead, they automatically used their implicit perception of the sequence set mean and range to respond positively to test elements that matched or were close to the set mean. Thus, performance was more accurate for test SEEN elements that equaled the mean–SEENmean–NEWin (see Fig. 2a–b, middle bars; Fig. 2c–d, left bars). When the NEW test element was equal to the set mean, it was frequently chosen as if it were a member (i.e., as if it had been seen in the set sequence). Participants actually chose this mean NEW element more frequently than the actual nonmean SEEN element—SEENin–NEWmean (see Fig. 2a–b, leftmost bars; note that accuracy below 0.5 means that the NEW element was chosen more frequently than the SEEN one.)

In addition, we found a range effect (i.e., participants rejected out-of-range nonmembers; SEENmean–NEWout and SEENin–NEWout) more frequently than in-range NEW test elements (SEENmean–NEWin, SEENin–NEWin, SEENin–NEWmean). This is shown in Fig. 2a–b, right two bars, and in Fig. 2e–f, right bars, compared with left bars in each graph. The same effect was seen for response time (RT; Fig. 2g), which was shorter for out-of-range than in-range NEW test elements, indicating they were rejected more rapidly as well as more frequently.

We concluded that participants automatically and implicitly determined the mean and range of the RSVP sequence even though they were not instructed to do so and even though this had no bearing on performance of the task at hand, which was just to try to remember the seen sequence elements. Furthermore, they did so on the fly for each trial, independently, since each trial had a different sequence mean and range.

Perception of set mean and range is not only implicit. In another study, Hochstein et al. (2018) asked observers to explicitly compare means of two arrays of variously oriented bars (mean comparison) or report presence of a bar with an outlier orientation among the array elements (outlier detection). It was found that mean comparison depended on the difference between the array means, and outlier detection depended on the distance of the target from the array range edge (see also Hochstein, 2016a, 2016b; Hochstein, Khayat, Pavlovskaya, Bonneh, & Soroker, 2018). Thus, both set mean and range are perceived both explicitly and implicitly.

The goal of the current study is to test whether there are identical effects in the related perceptual phenomenon of categorization.

## Experiment 1. Category prototype and boundary effects

### Prototypes as averages

We investigate here the nontrivial comparison between stimulus sets and object categories. The stimuli in previous studies of statistical perception were very similar, in each case, usually differing by a single varying feature (e.g., Ariely, 2001; Corbett & Oriet, 2011), or a combination of features forming a single high-level feature (e.g., facial expression; Haberman & Whitney, 2007, 2009). In contrast, categories might be thought of as a set of objects composed of combinations of multiple features, with only some of these features necessarily present in each category exemplar (where membership is defined by family resemblance). Thus, we compare the mean of the set elements with the prototype of category exemplars, based on the view that prototypes are the central or most common representations of a category (Goldstone & Kersten, 2003), possessing the mean values of its attributes (Langlois & Roggman, 1990; Reed, 1972; Rosch & Lloyd, 1978; Rosch, Mervis, et al., 1976; Rosch, Simpson, & Miller, 1976). Note, however, that comparing these perceptual procedures does not depend on this definition of prototype, or even on prototype theory itself. Comparing categorization with set summary perception is valid simply because in both cases several stimuli are perceived as belonging together, perhaps inducing the same response, because they share some characteristics and differ in others.

Similarly, we compare knowledge of category boundaries with perception of set range edges. As shown above, perceiving set range edges allows for rapid detection of outlier elements, and even unconscious perception of these edges allows for rapid rejection of out-of-range elements when trying to remember which elements were previously viewed. This was called the “range effect” (Khayat & Hochstein 2018). Similarly, knowing category boundaries allows for rapid separation of objects that belong to different categories, which we shall call a “boundary effect.” Thus, we compare properties of set perception and categorization in terms of observers’ implicit determination and knowledge of both the set mean and category prototype, as well as, the set range edges and the category boundaries. That is, having found that observers perceive rapidly and implicitly the mean and range of element sets, and that they use this information when judging memory of sequence stimuli, we now test if the same characteristics are present for object categories. Do observers of a sequence of objects determine automatically and implicitly their category and use the implied prototype (whether shown or not shown in the sequence) and the boundaries of the implied category, when later choosing images as having been seen in the sequence? These will be called the prototype and boundary effects, respectively. If we find similar characteristics in these processes, for categorization as for set perception, we will suggest that they may share basic perceptual-cognitive mechanisms.

We note at the outset that there are important differences between perceiving set summary statistics and categorizing objects. We perceive the mean size, orientation, brightness, and so forth, of sets that we see just once, sets which are unrelated to any other sets seen before. Presented with a set of images, sequentially or simultaneously, we derive the mean and range of the size, orientation, brightness, and so forth, of that set, on the fly and trial by trial. Thus, presented with a single stimulus in isolation, it is logically inconsistent to ask to what set it belongs. In contrast, by their very nature, categories are learned over a lifetime of experience, and with this knowledge, we can know immediately to what category a group of objects, or even a single object belongs. In fact, one of the defining characteristics of “basic” categories is that these are the names given to single objects (e.g., cat, car, fork, apple; Potter & Hagmann, 2015; Rosch, Mervis, et al., 1976). The situation with categorization is unlike that with sets, where we derive the set mean, on the fly, as we are presented with set members. Instead, when encountering an object (or group of objects belonging to a single category), we know the category to which it belongs, and we also know what is the prototype of that category and the category boundaries; there is no need, and no possibility, of deriving anew the category, prototype, and boundaries of a group of familiar objects (though we can learn new categories of unfamiliar objects; see Hochstein et al., 2019). Furthermore, categories may be learned and recognized semantically, while the basic features of sets are often nonsemantic. Nevertheless, and this is the basic argument of the current study, there may be similarities, if not identities, of mechanisms for representing set means/ranges and category prototypes/boundaries. We set out here to find the degree of similarity between these very different phenomena before endeavoring to uncover underlying mechanisms. Finding similarities, despite the differences enumerated above, would suggest that there are relationships between low-level and high-level representations of images, objects, categories, and concepts.

## Method

**Fig. 3:**
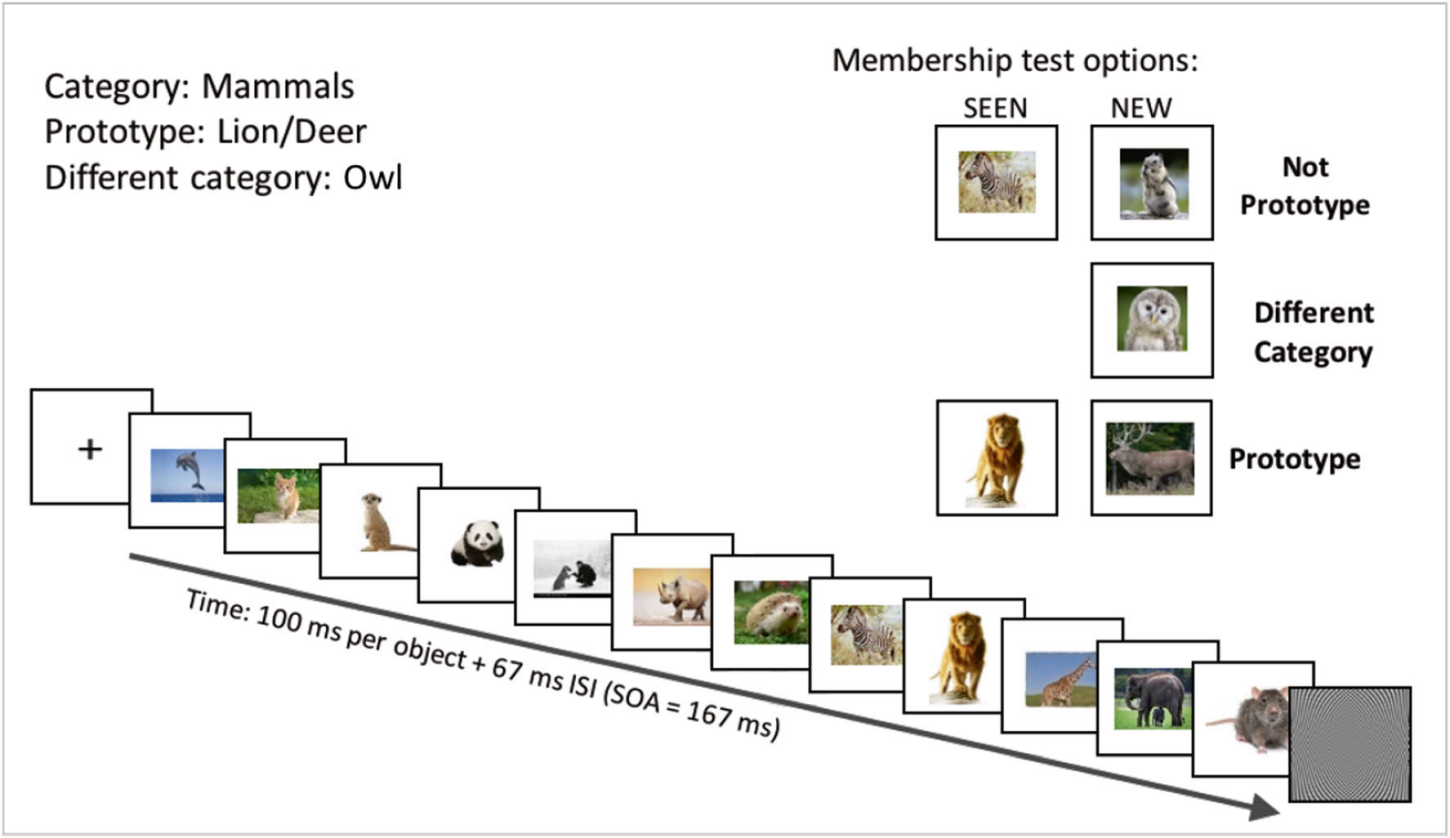
High-level category RSVP membership tests. Example RSVP trial with *mammals* as the set category. On the membership test, one of the optional subtype pair of images (see Table 3) was presented for the SEEN and the NEW images. The five trial subtypes for each of the 39 categories are designed by choice of the test images. A SEEN object image could be either SEENin (regular object image that was seen in the sequence and is a member of the category, not the prototype) or SEENprot (seen in the sequence and a prototype of the set category), while the nonmember object could be NEWin or NEWprot (object image from the same category but not included in the sequence, or the category prototype, again not presented in the sequence), and could also be NEWout (belong to a different category)

We present rapid stimulus visual presentation (RSVP) sequences of images of high-level category objects, conditions known to impair focused attention to each stimulus, but maintain statistical and categorical representations across time (Corbett & Oriet, 2011; Potter, Wyble, Hagmann, & McCourt, 2014). We then present two images, one identical to one of the images in the sequence (the SEEN image) and the other an image of a novel object (the NEW image). Observer task is to choose the SEEN image—the image that was present in the sequence. This is a two-alternative forced-choice (2-AFC) test, which is thus criterion free, and has a chance guessing level of 50% (see Fig. 3).

We do not inform observers that one of the imaged objects (either the SEEN or the NEW object) may be prototypical of the sequence category, and one (the NEW object) may be outside the sequence category (i.e., belong to another category). Note that when NEW objects were chosen from a different category, still, they were purposely chosen to be not too distant from the sequence category—that is, from a relatively close category (i.e., for basic level categories, a NEW object from the same superordinate category; all NEW objects from the same biological, nonbiological, or abstract concept groups of Table 2; for example, for the category mammal, a nonmammal animal; for dogs, a different mammal; for trees, another plant; for food, a drink; for weapon, a screwdriver; for toy, a sand clock). We hypothesize that the influence of prototypes on implicit categorization and thus on memory will be similar to the influence of the mean when we tested set item memory (Khayat & Hochstein, 2018). Thus, we expect observers to accept prototypical objects as SEEN more frequently (irrespective of whether they were in the sequence). Additionally, the presence in the test pair of an object outside the sequence category may aid in rejecting it as not seen in the sequence, and thus, NEW, just as items outside the set range were more easily rejected as NEW and not SEEN (see Fig. 2e–f).

**Table 2:** Categories with examples of their prototypes and other exemplars

Category level		Exemplar types	
Superordinate level	Basic level	Typical exemplars (Prototypes & Common)	Nonprototype exemplar
Plants*		Potted plant, Cactus	Watermelon plants, Vine
	Trees*	Oak, Olive	Sequoia, Baobab
Fruits*		Apple, Orange	Pomegranate, Litchi
Animals*		Dog, Deer	Mosquito, Octopus
	Reptiles	Python, Iguana	Legless Lizard, Commodore
	Birds*	Owl, Pigeon	Penguin, Pelican
Mammals*		Cow, Lion	Whale, Bat
	Dogs*	German Shepard, Labrador	Chi Wawa, Bull-Terrier
Food*		Pasta, Pancakes	Cake, Sushi
Weapons*		Pistol, Riffle	Cannon, Molotov bottle
Books		Harry Potter, The Bible	The Hobbit, Comics
Kitchen tools*		Whisker, Slicing Knife	Grater, Blender
Toys*		Teddy bear, Rubik’s cube	Top, Plastic food
Furniture*		Armchair, Sofa	Dresser, Stool
	Desks	Office desk, Writing desk	Reception desk, Cubicle desk
Houses		Villa, Apartments	Igloo, Canoe
Vehicles*		Car, Bus	Unicycle, Helicopter
	Cars*	Sedan, Hatchback	Formula 1, Model T
Liquids		Water, Milk	Acetone, Soap
	Drinks*	Milk, Beer	Cognac, Sake
Electronics*		TV screen, Laptop	Hair dryer, Shaver
Clothes*		Shirt, Trousers	Socks, Gloves
Games		Puzzle, Chess	Bowling, Super Mario
Music		Musical note, The Beatles	Mexican band, Accordion
	Sports*	Soccer, Basketball	Bowling, Billiards
Religion		Jesus, Western Wall	Buddha, Praying man
Science*		Test tubes, Atom	Lecture, MRI
Conflicts		Israeli–Palestinian	Random couple argument
Symbols		Peace symbol, David star	Scouts symbol, Recycle symbol
Occupations*		Judge, Policeman	Fisherman, Violinist
Disasters		9′11 Plane crash, Tsunami	Volcano eruption, Avalanche
Movies		The Godfather, Cinema & Popcorn	Cameraman, Script
	Horror	Wolf & full-moon, Hannibal Lecter	Scared face, Creepy doll
	Cartoons	Mickey Mouse, The Simpsons	Scooby-Doo, Hello Kitty
Events		Wedding, Festival	Graduation ceremony, Parade
Travel		Passport & Suitcase, Backpackers	Airport, Sunglasses
Health		Heartbeat icon, Workout	Nonsmoking, Granola
Hazard		Slippery sign, Toxic (skull) sign	Unstable bridge, Medusa
History		Martin Luther King, Hiroshima	Che Guevara, Mayan temples

*Note.* The 39 categories used in the student experiment; 20 categories for MTurks, indicated by *. Categories are placed in the first or second column according to their being superordinate or basic level categories

### Participants

Data of 15 in-house participants, students at the Hebrew University of Jerusalem, were included in the analysis of Experiment 1 (age range = 20–27 years, mean = 23.4 years; four males, 11 females). We also have results for 226 Amazon Mechanical Turk (MTurk) participants for Experiment 3. Participants provided informed consent and received compensation for participation and reported normal or corrected-to-normal vision.

### Stimuli and procedure

Procedures for Experiment 1 took place in a dimly lit room, with participants seated 50 cm from a 24-in. Dell LCD monitor. We have less information as to their identity and precise experimental conditions of the Experiment 3 Amazon MTurks (we excluded ~25% of these data for trials with RTs <200 ms or >4 s and for subjects with <33% remaining trials or <60% correct responses overall, thus including as many trials/subjects as possible, excluding data that are clearly not responses to the stimulus; e.g., Fabre-Thorpe, 2011). Stimuli were generated using Psychtoolbox Version 3 for MATLAB 2015a (Brainard, 1997). MTurk testing used Adobe flash. Images, chosen from the Google Images database, were presented against a gray background (RGB: 0.5, 0.5, 0.5).

Stimuli consisted of rapid serial visual presentation (RSVP) of a sequence of high-level objects or scene images presented in the center of the display, with a fixed size of 10.4-cm high × 14.7-cm wide, as demonstrated in Fig. 3 (see also examples of images in Fig. 8). Experiment 1 was divided into three blocks of 65 RSVP trials each, with a short break between them, to complete 195 trials total per participant; Experiment 3 had 60 trials total for MTurk observers; one session/participant.

A set of images (12 for in-house students; nine for MTurks) was presented in each RSVP sequence, with 167 ms stimulus onset asynchrony (100 ms stimulus + 67 ms interstimulus interval), and the sequence was followed by a 100 ms masking stimulus. Then, after 1.5 s, two images were presented side by side, simultaneously, for the membership test; one, an object image that was SEEN in the sequence, and one a novel, NEW object image. Sequence SEEN and NEW images were randomly placed to the left and right of fixation in the middle half of the width and height of the screen, and participants indicated position of the SEEN image by key press. Images remained present until observer response. Since participants tend to perceive and remember better early and late elements, known as primacy and recency effects, in general and specifically in summary representations (Hubert-Wallander & Boynton, 2015), we excluded from the test member images the first and last two RSVP sequence images.

Thirty-nine categories (20 for MTurks of Experiment 3) were included in the experiment (see Table 2), including manmade and natural objects (animate, inanimate, and plants), and abstract conceptual scenes from different category levels. Each category was repeated in each trial subtype (see below), with entirely different images for each trial. For each category, we chose the three images that seemed to us to be closest to prototypical, and used them in the three test subtypes including a prototype (as nonmember or as member versus nonmember same/different category). Of the 39 categories used for Experiment 1, 20 were later tested in Experiment 2, and only these were used in Experiment 3. For almost all the 20 categories, which were also tested in Experiment 2 (see below), high typicality was confirmed; we discarded data for the few discrepant images (<6% of trials). For the remaining 19 more conceptual categories, which were not tested in Experiment 2 (and not used in Experiment 3), we depended on examples from the literature (e.g., Iordan et al., 2016; McCloskey & Glucksberg, 1978; Potter, Wyble, Pandav, & Olejarczyk, 2010) and experimenter judgement for in-house student participants (who came from the same cohort as experimenter NK). Note that if we err and choose nontypical images as prototypes, this would add noise and reduce results’ significance; thus, the results themselves confirm our choice. For the entirely new MTurk tests, we used a different approach, depending on Experiment 2, as described below. We purposely chose both basic and superordinate categories, as well as conceptual categories, to broaden the potential impact of our results.

### Trial subtypes

Trial subtypes were defined by the nature of the two test image objects vis-à-vis the sequence category (as in the low-level tests; see the Introduction and Khayat & Hochstein, 2018). Each SEEN test image could be of an object from the RSVP sequence category (denoted SEENin) or the prototype of this category (SEENprot). The NEW test image could be of an object from the RSVP category (NEWin) or even its prototype (NEWprot), but, in either case, not actually presented in the sequence; alternatively, the NEW object image could be an image of an object from a different category (NEWout). Figure 3 illustrates these image types. Each pair of test images could be of one of five subtypes, listed in Table 3, (denoted SEENprot–NEWin, SEENin–NEWin, SEENin–NEWprot, SEENin–NEWout, or SEENprot–NEWout). Each subtype was tested for each category listed in Table 2.

**Table 3:** Member recall test trial subtypes

SEEN test image (correct)	NEW test image (incorrect)	Expected performance
SEENprot	NEWout	Best
SEENin	NEWout	Better
SEENprot	NEWin	Better
SEENin	NEWin	Baseline
SEENin	NEWprot	Worse

*Note.* Each trial sequence of objects of a single category was followed by a pair of images of two objects, one a repeat of one of the object images in the sequence, the SEEN image, and one an image of a NEW object. Choice of the SEEN image is correct, of the NEW image, incorrect. Test pairs of subtype SEENin–NEWin have both SEEN and NEW objects from the sequence category (“in”), but neither is the prototype. This is the baseline subtype against which results from the other subtypes will be compared. In subtype SEENprot–NEWin, the SEEN object is the category prototype, and the NEW object is a category exemplar not shown in the sequence. If memory of the prototype is easier, we expect better performance for this subtype than for subtype SEENin–NEWin. In subtype SEENin–NEWprot, the NEW image object is the category prototype, which was *not* shown in the sequence, and the SEEN object is not the prototype. If there is “false memory” (i.e., after seeing a sequence of objects of a particular category, observers “recall” having seen the category prototype), then they might choose, incorrectly, the unseen prototype rather than the seen object image. In subtypes SEENprot–NEWout and SEENin–NEWout, the NEW object image is from another category (“out”), and the SEEN object is either the prototype (SEENprot) or is not the prototype (SEENin). Here, we expect easy rejection of the NEW image object because it is of a different category. Irrespective of trial subtype, participants sometime choose the SEEN test image because they remember seeing it in the sequence. Trial subtypes were presented in randomized order without observers knowing about this classification

### Statistical tests *and data analysis*

Analysis of variance (ANOVA) tests with repeated measures were conducted to verify that performance accuracy differences were due to the difficulty derived by effects emerging from the different trial subtypes, rather than within-participant differences in performance. For the two-way repeated-measures ANOVAs, testing student participant effects of SEEN object typicality and NEW object category, we combined data for NEW object same category, whether prototypical (NEWprot) or not (NEWin); *t* tests (one-tailed) between the averaged results of all participants for different subtype combinations were performed to investigate prototype and boundary representations effects. Since it is difficult to remember all the sequence images, we expect participants to correctly prefer as SEEN those test images with objects that are prototypes of the sequence category (expected fraction correct for SEENprot–NEWin > for SEENin–NEWin) and mistakenly choose the NEW test image when it is the category prototype, though not seen in the sequence (expected fraction correct for SEENin–NEWin > for SEENin–NEWprot), and to reject, as seen in the sequence, those that are of a different category (fraction correct for SEENprot–NEWout > for SEENprot–NEWin; and SEENin–NEWout > SEENin–NEWin).

## Results

**Fig. 4:**
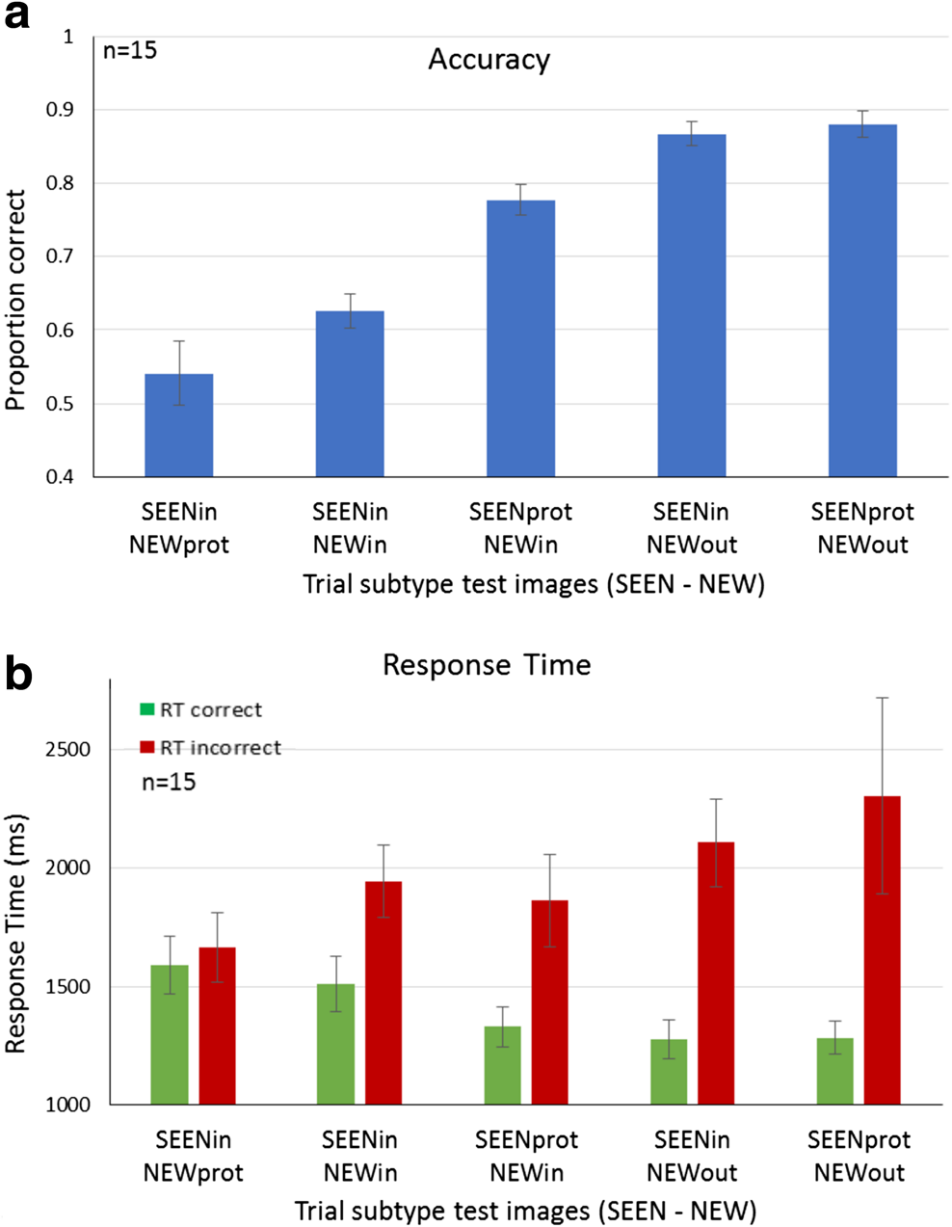
High-level image memory performance by RSVP trial subtype (Students). **a** Accuracy rates sorted by test image subtype (SEEN = object image seen in trial sequence, NEW = object image not seen in trial sequence). **b** Response time measured for correct (choice of SEEN image; green) and incorrect (choice of NEW image; red) responses, sorted by test image subtype. (Color figure online)

**Fig. 5:**
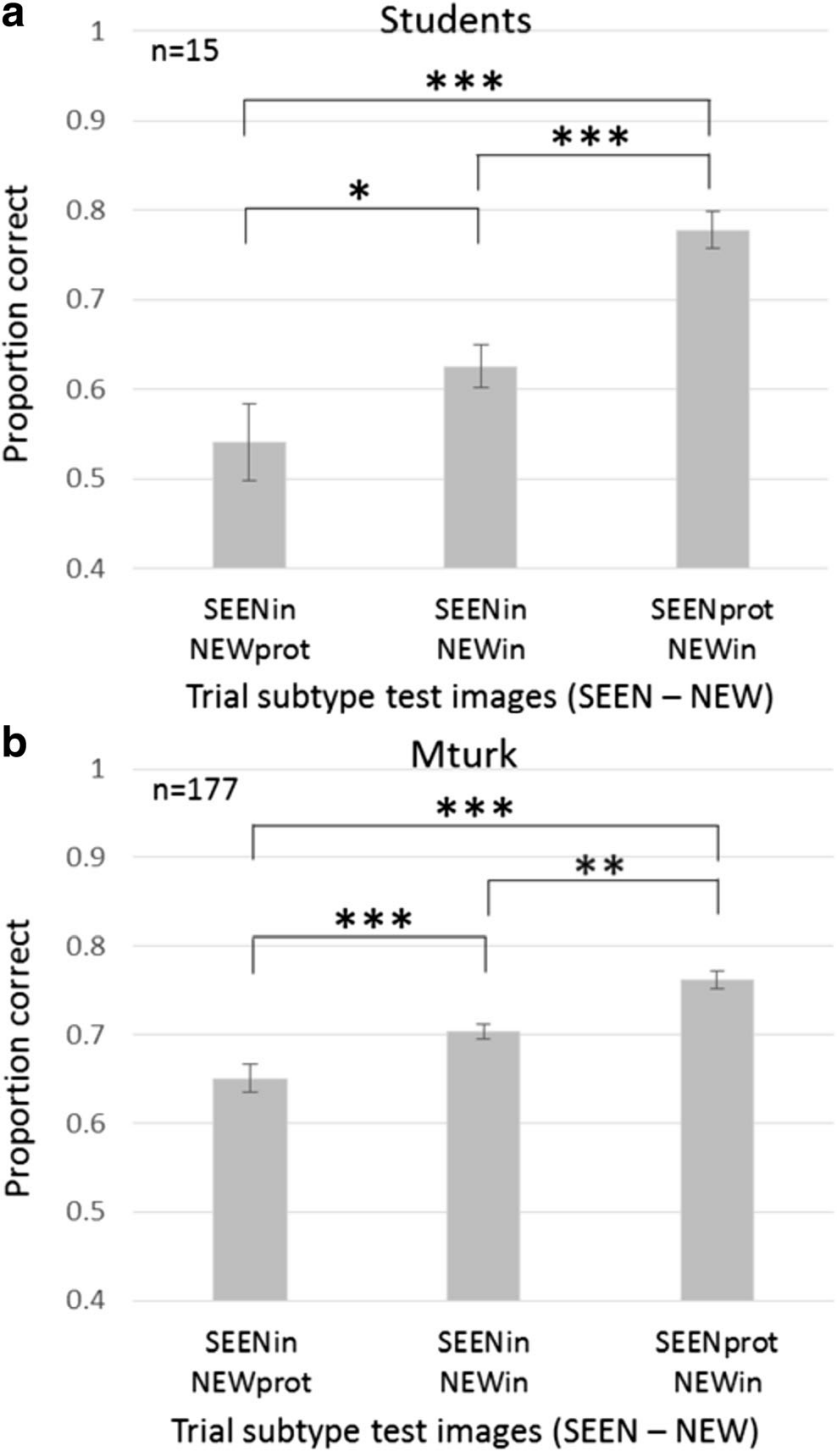
Category prototype object effect on accuracy. Proportion correct for those subtypes for which both test objects are within the sequence category: SEENin–NEWprot, SEENin–NEWin, and SEENprot–NEWin; *t* tests among the subtypes show significant differences, indicating the expected prototype effect on observer judgment in membership tests, with a preference to choose the object which matches the category prototype (SEEN = object image seen in sequence, NEW = object image not seen in sequence). **a** Students. **b** MTurks. Significance indicated by *p < .05. **p < .01. ***p < .001

The two basic measurements indicating observer performance are accuracy rates and response time (RT) for each trial subtype, as shown for student participants in Fig. 4. The results by trial subtype roughly resemble those from the low-level experiment, demonstrated in Fig. 2b, with some effects even more salient, as detailed below. Figure 5 presents averaged accuracy results across participants, sorted by subtype, isolating the three subtypes with both test image objects within the sequence category (subtypes SEENprot–NEWin, SEENin–NEWin, SEENin–NEWprot), for student (Fig. 5a) and MTurk participants (see Fig. 5b).

We performed a two-way repeated-measures ANOVA on the Fig. 4 results. The overall prototype effect—the effect of one of the objects being the prototype of the category of the objects presented in the sequence—was significant, *F*(1, 14) = 18.07, *p* < .001; the boundary effect—the effect of the nonmember being of another category than the sequence objects—was highly significant, *F*(1, 14) = 298.64, p < .001, and the interaction between them was significant, as well, F(1,14) = 13.36, *p* < .005. The interaction effect suggests that the prototype effect may be larger in some cases, as we shall see in the following paragraph.

### Prototype effect

The first factor to influence performance is the presence of category prototypical objects (prototypes and most common or familiar objects) in one of the test images. The presence of typical exemplars influenced accuracy (% correct responses) and RT, which together we call the prototype effect. As seen in the three left bars of Fig. 4a and 5a–b, prototype presence affected accuracy: accuracy SEENprot–NEWin > SEENin–NEWin > SEENin–NEWprot. Prototype presence also affected response time (RT), as in Fig. 4b: RT correct choice of member SEENprot–NEWin < SEENin–NEWin; RT incorrect choice of nonmember SEENin–NEWprot < SEENin–NEWin).

It is possible that when including subtypes with NEWout test images (i.e., images of an object of a different category; subtypes SEENin–NEWout and SEENprot–NEWout) in the above two-factor ANOVA calculation, the effect of the presence of a different category (NEWout) reduces the prototype effect. Thus, to test the prototype effect alone, we conducted a one-way repeated-measures ANOVA on the three subtypes, with test image objects *in* the category boundaries (see Fig. 5). This one-factor ANOVA showed a significant prototype effect—students: *F*(2, 28) = 11.78, *p* < .001; MTurk: *F*(2, 346) = 26.96, *p* < .001. We conclude that, as predicted, when comparing trials containing only objects from the relevant category (subtypes SEENprot–NEWin, SEENin–NEWin, SEENin–NEWprot), the prototype had a major influence on observer response, which tended to attribute it as a member of the RSVP sequence regardless of whether it was or was not.

On the other hand, there is no significant difference between the case where the SEEN image object is prototypical or not when the NEW object is outside the category (accuracy for SEENprot–NEWout = 0.88 versus for SEENin–NEWout = 0.86; *p* = .59; see Fig. 4a). The boundary effect overrides the prototype effect (leading to the interaction effect in the two-way repeated-measures ANOVA, above).

We conclude that, due to limited attentional resources, participants are unable to fully perceive and memorize all individual objects, but still succeed in having a good representation of the category itself. This is striking, since the stimuli were presented in RSVP manner, with brief periods between stimuli. Nevertheless, observers were able to detect the sequence category and derive its prototype. They were successful in both category and prototype determination for sequences that included basic level, subordinate, superordinate, or even conceptual categories. They tend to relate the most representative object (the prototype) to the category of the presented object images and assume it was present in the sequence (see Fig. 5a: students; Fig. 5b: MTurks). We performed post hoc *t* tests between the different subtypes to find details of the effect, as shown in Fig. 5a–b. The prototype effect is clearly present when comparing the relevant trial subtypes (SEENprot–NEWin, SEENin–NEWin, SEENin–NEWprot), which significantly differ from each other (students: *p* < .05 for subtypes SEENin–NEWin versus SEENprot–NEWin or SEENin–NEWprot and *p* < .01 for SEENprot–NEWin versus SEENin–NEWprot; MTurks: *p* < .001 for all comparisons). These subtypes create a staircase shape from low performance of 0.54 ± 0.04 (MTurk: 0.64 ± 0.01; mean ± *SE*) proportion correct for SEENin–NEWprot, via 0.63 ± 0.02 (0.7 ± 0.008) correct for SEENin–NEWin, to best performance of 0.78 ± 0.02 (0.76 ± 0.01) correct for SEENprot–NEWin. We ask below if this is an all-or-none prototype-or-not-prototype effect, or if it is a graded effect, as objects are more or less typical of the category. Note that, surprisingly, even when the prototype was not present in the object sequence, it was often chosen as present when presented as the NEW test image. Nevertheless, when choosing between a nonprototypical SEEN image and a prototypical NEW image (SEENin–NEWprot), having actually seen the image in the sequence is slightly more important than typicality (0.54 and 0.64 for students and MTurks, respectively; significantly > .50). This is different than the results found for the low-level feature set, as is easily seen in the proportion correct for the SEENin–NEWprot subtype (>.5) compared to the analogous SEENin–NEWmean subtype (<.5). We believe that the difference derives from the greater observer memory for images of real objects, compared to memory of absolute values of simple features of abstract images (circle size, line orientation, disc brightness).

**Fig. 6:**
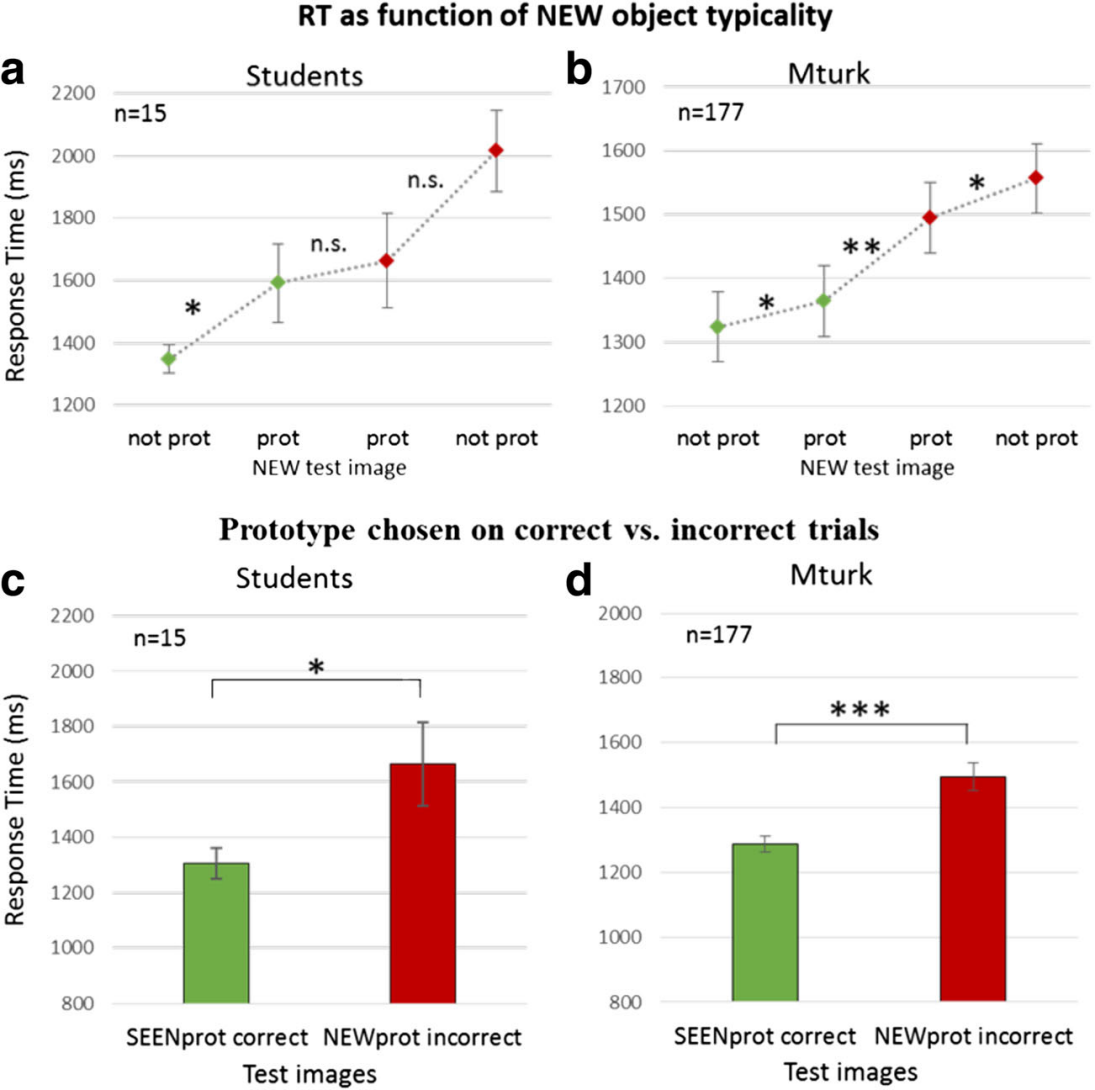
Response Time Prototype Effect. **a** Students: RT for each combination for the NEW test element as prototype, not prototype, correct, and incorrect trials. Green and red diamonds represent correct and incorrect trials, respectively. Left: RT compared for correct trials where the NEW test image object is the prototype of a category (SEENin–NEWprot) versus all other trials where it is not the prototype. Right: RT compared for incorrect trials where the NEW test image is of the prototype of a category (SEENin–NEWprot) versus all other trials where it is not of the prototype. Middle: RT compared for the NEW object being the prototype and participants choosing this image, incorrectly, or the nonprototype SEEN image, correctly. **b** Similar graph for MTurk participants. **c** Students: RT comparison between trials with participants picking prototype object images correctly (green bar) versus incorrectly (red bar). **d** Similar graph for MTurk participants. ***p = .001. (Color figure online)

We conclude that with a failure of coding all the individual sequence images, due to brief image exposure times, the presence of prototype object images had a significant effect on the responses, whether they were seen or new images of the RSVP category. Along with these accuracy differences, an analysis of the response times (RT) provides additional support for the conclusion that participants perceive prototypes as ideal representatives of the category and “remember” these whether they were present or not. In Fig. 6a–b, RT is classified into trials in which the NEW test image is correctly rejected (Fig. 6a–b, left green) or, incorrectly, chosen (Fig. 6a–b, right red) comparing when the NEW object either is or is not a prototype. As expected, Fig. 6a–b shows that correct responses (green) are made faster than incorrect responses (red), like the comparisons seen in Fig. 4b. The details show further interesting comparisons, as follows. Analysis of the correct RTs indicate that when participants did correctly chose the nonprototype SEENin test image, they did so significantly slower when the NEW image was a prototype (students: 1591 ms ± 125 ms; MTurk: 1364 ms ± 28 ms) than when the NEW image was not a prototype (students: 1348 ms ± 46 ms; MTurk: 1319 ms ± 23ms; *t* test *p* < .05), as displayed in Fig. 6a–b, left diamonds. In other words, not only were they often manipulated to falsely pick the prototype as having been seen in the sequence (see Fig. 5), even when they did manage to choose a nonprototype SEEN image, their response was delayed, as if the presence of the NEW image being a prototype (SEENin–NEWprot) affected their confidence. In addition, choosing the correct SEEN object is faster when it is the prototype (SEENprot–NEWin versus SEENin–NEWin and SEENin–NEWprot, see Fig. 4b, left three green bars). Furthermore (Fig. 6a–b, right red), choosing the NEW object, incorrectly, is faster when it is the prototype than when it is not (students: 1663 ms ± 150 ms versus 2015 ms ± 130 ms; *t* test: *p* = 0.174, *ns*; MTurk: 1495 ms ± 41 ms versus 1557 ± 31 ms; *p* < .05).

On the other hand, besides the prototype effect, there is still some degree of recognition of test objects having been seen in the sequence. Thus, as demonstrated in Fig. 6c–d, choosing the prototypical object is faster when it is a sequence member (correct: SEENprot–NEWin; and SEENprot–NEWout for students; students: 1304 ms ± 50 ms; MTurk: 1288 ms ± 25 ms) than when it is not (SEENin–NEWprot incorrect; students: 1663 ms ± 150 ms; MTurk: 1495 ms ± 41 ms; *t* test: *p* < .05, *p* < .001, respectively). Even choosing the nonprototypical seen image is faster than choosing the typical new image (see Fig. 6a–b, middle two diamonds; *t* test: *p* = .061, *p* < .01). This latter speed joins the greater accuracy (see above) to indicate it is not a speed–accuracy trade-off.

### Range/boundaries effect

**Fig. 7:**
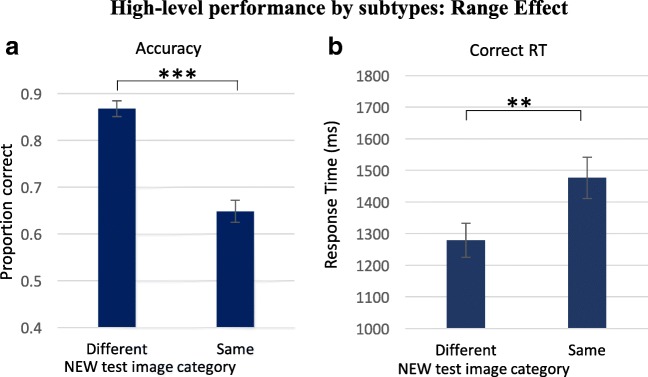
Range (category) effect—within versus between category differentiation (students). **a** Average accuracy for subtypes SEENin–NEWout and SEENprot–NEWout versus subtypes SEENprot–NEWin, SEENin–NEWin, and SEEN–NEWprot. Observers were more accurate when the nonmember test object was from a different category than that of the RSVP sequence. **b** RT of correct trials was significantly faster when the nonmember object belonged to a different category than when both test objects belonged to the RSVP sequence category. **p = .01

The second statistic found for low-level sets is the range effect, whose equivalent would be representation of category boundaries. A two-way repeated-measures ANOVA was performed on accuracy and revealed a highly significant boundary effect, as shown above, *F*(1, 14) = 298.64, *p* < .001. As with low-level features, accuracy rates in trials of nonmember objects outside of category boundaries (SEENprot–NEWout and SEENin–NEWout; i.e., NEW objects from a different category than the object sequence, were significantly higher, 0.87 ± 0.02, than in trials with both test objects within the category range, SEENprot–NEWin, SEENin–NEWin, SEENin–NEWprot; 0.65 ± 0.02; *p* < .001), as seen in Fig. 7a.

This effect was observed also in response time measurements for correct responses, as shown in Fig. 7b. Responses were significantly faster for trials where the nonmember object was outside category boundaries (i.e., belongs to a different category, 1279 ms ± 54 ms), than in trials where both test objects were from the category of the RSVP sequence (1476 ms ± 65 ms; *p* < .01). Taken together, the increase in accuracy and decrease in RT indicate a consistent trend of reducing task difficulty by introducing nonmember test objects from a different category, rather than a speed–accuracy trade-off.

## Experiment 2. Scoring object typicality

So far, we have compared results for category and set sequence member recall and effects of prototype—mean and boundaries—range edge on choice of member image in a 2-AFC task. In addition, Khayat and Hochstein (2018) measured how these mean and range effects are graded with the distance of the test item from the mean or from the range edge. To complete and quantify the comparisons, we would like to do the same for the prototype and category effects seen here. To this end, we need a measure of the distance of our test objects from their category prototype. (It would be nice to measure how far away from a category are objects from different categories, but this seemed too difficult for the present study.)

The current experiment was therefore designed to measure the subjective distance of objects from their category prototype, and to learn for each category which object is the prototype itself. To this end, we asked 50 MTurk participants to choose one of two image objects as a member of a previously named category, and used their response speed as a measure of the closeness of the object to the prototype. We will then use these results in Experiment 3 to measure the graded prototype effect. It has been well documented that responses are faster for prototypes than for non-prototypes (Ashby & Maddox, 1991, 1994; McCloskey & Glucksberg, 1979; Rips, Shoben, & Smith, 1973; Rosch, Simpson, & Miller, 1976). We note in the Discussion that responses may also be faster for more familiar objects, and that there is debate concerning the relationship between familiarity and typicality.

## Method

### Stimuli and procedure

We present the name of a category in the middle of the screen for 1s, (font: Arial 32, white), followed, after 1.0 s, by two test images, one of an object belonging to the named category, and one of a different category (attempting to choose objects that were from a different category but not too far from the named category; see Experiment 1, Method section). Images were presented to the left and right of the center of the display, in the middle half of the width and height of the screen. Images remained present until observer response.

Observer task was to choose, by key press, the image with an object that belongs to the named category. We hypothesize that the closer the object is to the category prototype, the faster will be the response, expecting participants to recognize prototypical objects as members of the named category quicker than they do atypical members. For example, participants will recognize an apple as a fruit faster than a kiwi, a cow as a mammal faster than a dolphin, and baseball as a sport faster than mountain climbing.

We tested 50 Amazon Mechanical Turk participants (MTurks). Participants performed two sessions of 300 trials/session. They were tested on 20 categories, as indicated in Table 2 (starred categories), 10 categories per session, with 30 test objects for each category.

## Results

As expected, response times varied among objects (maximum: 2.04 s; minimum: 0.65 s; mean range for 20 categories: 0.65 s), and there was significant correlation among participants (mean standard error between participants was 6% of the RT).

**Fig. 8:**
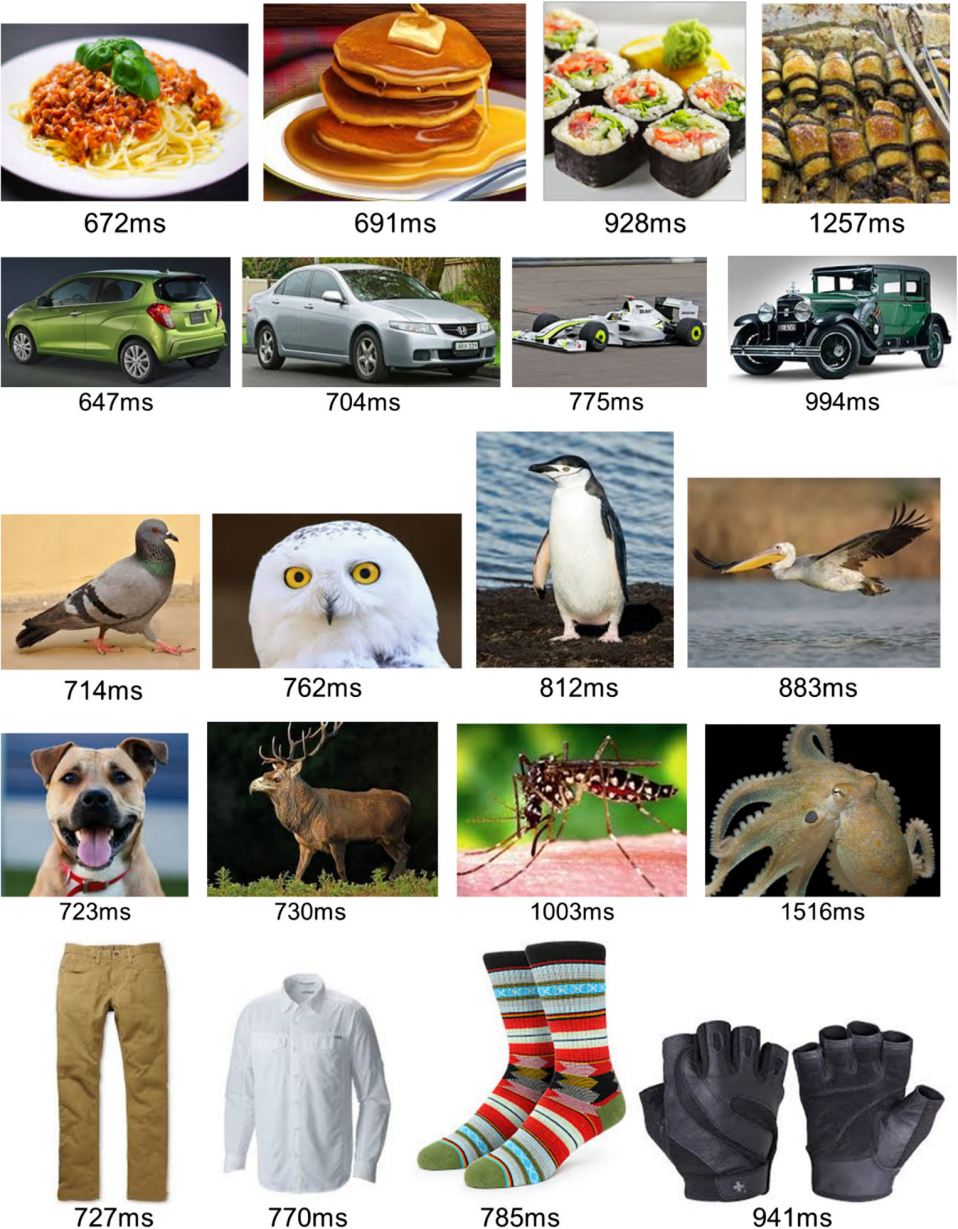
Examples of category objects and their associated response times. Four example objects are shown for each of the categories of food, cars, birds, animals, and clothing, with the mean RT over 47 observers. We assume that shorter RTs are associated with objects that are closer to the prototype, and use the RT ranking of objects for each category as a measure for its typicality

Examples of categories and their objects are shown in Fig. 8. For each category, four objects are shown, and for each, the mean RT was measured for our 50 MTurk observers.

**Fig. 9:**
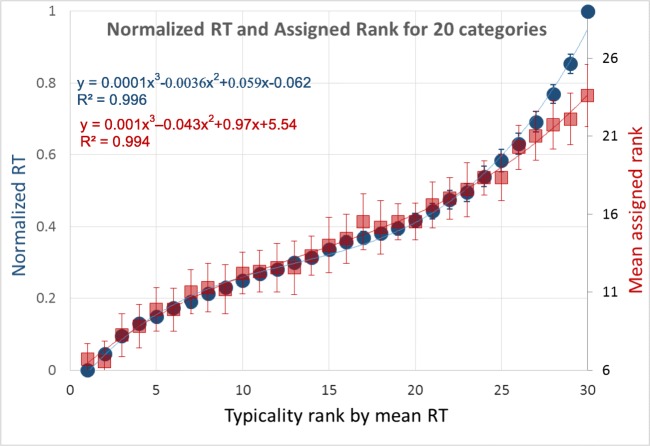
Average normalized RT for each category object rank. Objects were ranked from 1 to 30 for each category according to the RT in the scoring object typicality test, where observers simply indicated which of two objects belonged to a previously named category. We then normalized the actual RTs, averaged over participants, and compare the result with the ranking (blue). The fit of the two measures is very good, with good agreement among participants. Also shown is the mean and standard deviation across participants of the rank assigned to each objects (red). The results match closely the mean RT data, and the across standard deviation is small, confirming the methodology. (Color figure online)

We ranked the objects of each category (from 1 to 30) and computed the mean RT for each rank over all 20 categories. These average RTs were then normalized by: Normalized RT = (RT − minRT) / (maxRT − minRT), where minRT and maxRT are the minimum and maximum RTs for that category, and (maxRT − minRT) is the range of average (across participant) response times for each category. Figure 9 (blue symbols) demonstrates the average normalized RT for each category object rank. There is a high degree of across-category similarity, evidenced by the small standard error among the categories. Interestingly, RT dependence on rank is steeper at the edges of the category objects, near the prototype (rank = 1) and far from it (rank = 30). We also measured the across-participant ranking and found small standard deviations (see Fig. 9, red symbols). We shall now use this ranking as a typicality index for each item in its category, to measure the impact of typicality on object memory in the RSVP sequence test.

## Experiment 3. Graded typicality

**Fig. 10:**
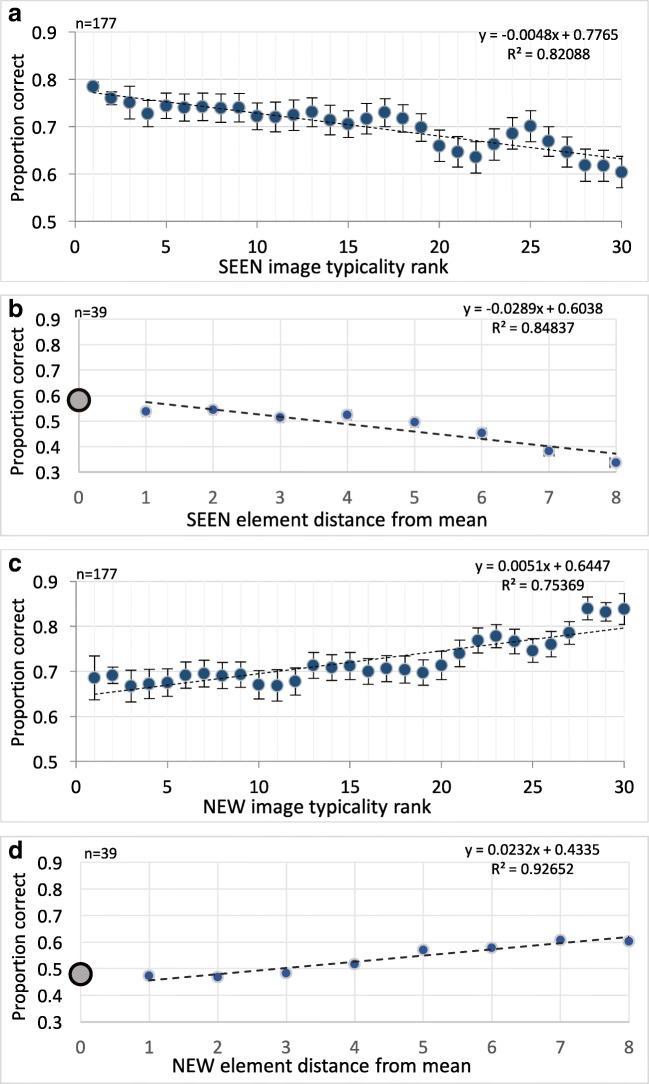
Graded prototype effect. **a** Proportion correct as a function of the typicality index of the member test object where typicality is ranked from 1 to 30 (1 closest to prototype). **b** Similar graph for low-level feature experiment (from Khayat & Hochstein, 2018). Proportion correct as function of member test-element distance from set mean. Note similar gradual decrease in probability of choosing the member as it is further from the prototype/mean. **c** Proportion correct as a function of the typicality index of the nonmember test object as it is gradually further from typical, so that this object is more easily and more often rejected (i.e., less often chosen as the sequence member). **d** Similar graph for low-level feature experiment. Note similarity between low-level feature and high-level categorization effects

Having derived a measure of the distance of each object from its category prototype—the typicality index—we now use this index to measure the impact of typicality on memory of objects in a previously seen sequence. For low-level objects (Khayat & Hochstein, 2018), it was easy to measure the distance of each element from the mean of the sequence since the elements differed by a measurable feature (orientation, brightness, size; see Fig. 1b). We found there, as shown in Fig. 10b and d, that the mean effect is graded. That is, as the member element is closer to the mean, so it is preferably chosen as the member (see Fig. 10b). Similarly, as the nonmember is further from the mean, so it is rejected as not being the member (see Fig. 10d). We now ask if this same rule applies to category objects. We have seen the prototype effect in Fig. 5 as a preference to choose as the member objects that are exactly the prototype of the category. Is this effect also graded?

For Experiment 3, we tested MTurk participants (see Experiment 1, Method section) with the 20 starred categories in Table 2 and tested in Experiment 2. We use the mean across-participant RT found in Experiment 2 as the basis for the typicality ranking of objects for Experiment 3. Note that different MTurk participants were tested in Experiments 2 and 3 (Experiment 1 was with in-house student participants). For Experiment 3, all objects presented in the test pairs were from the same category as the previously presented sequence (only bottom three subtypes of Table 3), so that we are now testing the graded prototype effect, and not the range effect (seen in Experiment 1; Figs. 4 and 7).

## Results

Figure 10 displays the graded prototype effect. We measure the proportion correct, which is the probability of choosing the member object as having been seen in the category sequence, as a function of the typicality index of the member object (see Fig. 10a). Typicality is ranked from 1 to 30, where 1 is the closest to the prototype (i.e., the shortest average RT measured in Experiment 2). Note the gradual decrease in choosing the member as it is further from the prototype. Similarly, as the nonmember is gradually further from typical—that is, the mean RT to this object was greater in Experiment 2, so this object is more often rejected, and is less often chosen as the member (see Fig. 10c).

**Fig. 11:**
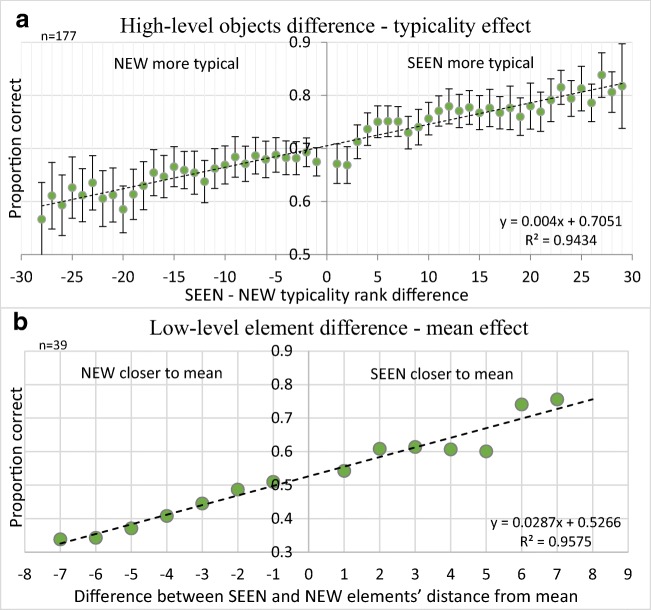
**a** Accuracy as a function of the difference between the distances of the nonmember and the member objects from typicality (considering that correct choice in the membership test depends on both the member and the nonmember image distances from the prototypical. **b** Parallel graph for low-level features (Khayat & Hochstein, 2018)

Despite the Experiment 2 nonlinear dependence of typicality rank on image RT, Fig. 10a and c data fit well a linear regression. This may be because of the near linearity of the Fig. 9 curve, except at its extremes, and because Fig. 10a averages over nonmember rank, Fig. 10c over member rank, and Fig. 11a over both.

The choice of an image is not dependent only on that image, however, since there are always two images displayed and we ask participants to choose between them. Thus, the relative measure between the two images should determine which image participants choose. Having found that sequence member object closeness to the prototype and sequence nonmember distance from the category prototype both add to correct choice of the member, we now plot choice accuracy as a function of the difference between the distances of the nonmember and the member. This is shown in Fig. 11a, where we also show the parallel graph for low-level features (Fig. 11b; from Khayat & Hochstein, 2018).

These graphs, including the high-level categorization graphs, are not without noise. Noise comes from the random second image in the membership tests, from interparticipant differences, and from the very nature of our using RT as a determinant for typicality. Nevertheless, the good fit to a single trendline suggests that our conclusion is well founded, as follows. When viewing a sequence of objects belonging to a single category, observers often fail to recall the identity of each object seen, and instead, when asked which of two objects was included in the sequence, depend, on recognition of the category seen, knowledge of the prototypical object, and estimation of the distance of the two test objects from the category prototype.

## Discussion

The current results confirm and extend those of recent studies suggesting that statistical representations generalize over a wide range of visual attributes, from simple features to complex objects, giving accurate summaries over space and time (Alvarez & Oliva, 2009; Ariely, 2001; Attarha & Moore, 2015; Chong & Treisman, 2003; Gorea et al., 2014; Haberman & Whitney, 2009; Hubert-Wallander & Boynton, 2015). This result is now extended to object categories, as well. These efficient representations overcome severe capacity limitations of perceptual resources (Alvarez & Oliva, 2008; Robitaille & Harris, 2011), and they are formed rapidly and early in conscious visual representations (Chong & Treisman, 2003), without focused attention (Alvarez & Oliva, 2008; Chong & Treisman, 2005) and without conscious awareness of individual stimuli and their features (Demeyere, Rzeskiewicz, Humphreys, & Humphreys, 2008; Pavlovskaya, Soroker, Bonneh, & Hochstein, 2015). Thus, their underlying computations play a fundamental role in visual perception and the rapid extraction of information from large and complex sources of data. In particular, we propose that categorization mimics set summary statistics perception processes that share its characteristics. Note that rapid gist perception does not imply low cortical level representation—on the contrary, it is the result of rapid feed-forward computation along the visual hierarchy (Hochstein & Ahissar, 2002).

Regarding high-level categories, we revealed two phenomena that match those found for low-level features, by using a similar experimental design for the two experiments: an RSVP sequence followed by a 2-AFC experiment test of image memory.

(1) Typicality effect: The typicality level of an object was well represented, as it biased participants’ decision toward choosing the more typical exemplar (of the presented category) as the member of the RSVP sequence. The typicality effect led to faster and more accurate responses for member test items, and also to choice of the incorrect item, when it had superior typicality (see Figs. 4–6 and 10–11). Thus, the more typical object was chosen as present in the sequence, whether it was or was not actually present there. The typicality effect is similar to the set mean value effect found for low-level features. (2) Boundary effect: Categorical boundary representation assisted participants in rejecting images with objects that do not belong to the category of the RSVP sequence; they therefore correctly chose the member image and achieved higher performance levels in these trials (see Figs. 4 and 7). This effect is similar the set range edges effect.

Furthermore, using a dedicated response-time test to rank the typicality of items within their category, we find that the typicality effect is graded, similar to the set mean value effect (see Figs. 10 and 11). The degree to which observers preferentially choose category items as having been members of the trial sequence is directly related to the degree of typicality of the test items. Both member and nonmember items are chosen more frequently as they are closer to prototypical; member items, correctly, and nonmember items, incorrectly. In particular, the relative typicality of the member test item versus the nonmember test item strongly affected observer choice of which item they reported as member of the sequence (see Fig. 11). Participants associated the more typical object to the displayed RSVP sequence, regardless of whether the prototype actually was or was not a member of the set. It is as if when viewing the sequence of objects, they perceived the category, but had only a poor representation of its individuals. This is exactly what was found for set perception (Khayat & Hochstein, 2018; Ward, Bear, & Scholl, 2016; but see Usher, Bronfman, Talmor, Jacobson, & Eitam, 2018).

We propose that participants unconsciously considered prototypes as better representatives of the categories than less typical exemplars and correspondingly chose them as members of the sequence, perhaps because prototypes usually contain the most common attribute values shared among the category members (Goldstone & Kersten, 2003; Rosch & Mervis, 1975).

As in the low-level experiment, participants were not informed about the categorical content of the RSVP sequences, and so they had no knowledge concerning the involvement of prototypes, categories, and so forth, and they only followed the instructions of an image memory task. The similarity of the effects emerging from the two experiments implies that statistical and categorical representations are cognate phenomena that share perceptual characteristics, and perhaps are generated by similar computations.

Note that both the category prototype and boundary effects are based on participants’ implicit categorization, extracted from the images in the RSVP sequences.

The results indicate that they adjusted their responses toward the relevant category, even though they were not guided to take category information into consideration in the alleged memory test. While participants concentrated on the RSVP images themselves, it seems that category context extraction overcame the cognitive abilities of memorizing the objects or scenes presented by the images.

Nevertheless, we note that accuracy in this experiment was superior to that in our previous set summary statistics experiment (compare Figs. 4 and 2; Khayat & Hochstein 2018). This may well be due to accurate memory of some sequence items, which is easier for object images than for abstract items (circles, disks, or line segments), which differ only in size, brightness, or orientation. This result also confirms that participants are trying to recall the actual objects displayed in the sequence—they sometimes succeed in remembering them—and they are not consciously trying only to categorize the images.

Categorical perception is often influenced by context (Barsalou, 1987; Cheal & Rutherford, 2013; Joubert, Rousselet, Fize, & Fabre-Thorpe, 2007; Koriat & Sorka, 2015, 2017; Roth & Shoben, 1983). Water, for example, may be associated with different categories, depending on context. It is a drink, a liquid for bathing or cleaning, or the medium of marine animals. Thus, the category to which participants associated each sequence object would naturally be affected by other sequence objects. We conclude that the current categorization processes occurred rapidly and intuitively, based on the variety of sequence objects, but also on earlier processing of interactions between objects and their contexts (Barsalou, 1987; Joubert et al., 2007; Koriat & Sorka, 2015, 2017; Roth & Shoben, 1983).

### Differences between low-level parameter sets and high-level categories

There are several differences between the low-level and the high-level results that should be pointed out. For the low level, we measured not only the graded mean effect, but also the graded range effect (i.e., the gradual effect of the distance of the presented nonmember element from the edge of the range of the presented sequence). This range effect has its equivalent in the boundary effect seen in Fig. 4. To extend this to a graded effect would require measuring the distance between an object of one category from the “edge” of a different category. This is beyond the scope of the current study.

A second difference to be noted is that it is easier to remember particular pictures of objects than specific elements in a sequence that differ only in a low-level feature (orientation, size, or brightness). Thus, as mentioned above, performance in the high-level test is superior overall. (Note performance axis difference between Figs. 10a, c and b, d.)

Another significant difference between testing the low-level set features and the high-level category objects is that the set of low-level elements, and their range and mean, are determined on the fly for each trial, by the sequence of stimuli actually presented. In contrast, the high-level categories are, of course, learned from life experience, and their prototype and boundaries are known immediately when seeing the first object in the sequence (or first few if the category is ambiguous). Categorization is thus predetermined, and not a result of the experience in the experiment itself. At the same time, there may well be interparticipant differences in the way they categorize objects, and, in particular, in the specific objects that they consider prototypical.

Related to the latter two differences is another. Categories are often denoted and remembered by their name, introducing a semantic element to the association of a variety of objects to a single category. This is not so for the low-level features studied previously. Nevertheless, recall that the world contains, naturally and intrinsically, objects that cluster separately in feature space, and thus categories that are language independent (Goldstone & Hendrickson, 2010; Rosch, Mervis, et al., 1976).

### Implications for categorization processes

There is ongoing debate concerning category representation in terms of the boundaries between neighboring categories, in terms of a single prototype (category members resemble this prototype more than they resemble other categories’ prototypes), or in terms of a group of common exemplars (new objects belong to the same category as the closest familiar object). Our finding that participants respond on the basis of both the mean and range of sets, and similarly on the basis of the prototype and boundary of object categories may suggest a hybrid categorization process model.

Concerning the single prototype versus multiple exemplar theories, our results may support prototype theory, since we find that participants choose test objects that are more prototypical, rather than recalling viewed exemplars. Nevertheless, category prototypes may be a secondary readout of fuzzy representations of multiple exemplars (see below).

We believe that the parallel found between set summary perception and perception of categories suggests there might be a common representation mechanism. We suggest that a population code (Georgopoulos, Schwartz, & Kettner, 1986) might underlie set representation of mean and range, and the same may be true for category prototype and boundaries (Bonnasse-Gahot & Nadal, 2008; Nicolelis, 2001; Tajima et al., 2016).

Observers clearly perceive not only the category of the sequence objects but also their typicality (compare Evans & Treisman, 2005; Potter et al., 2010). Furthermore, Benna and Fusi (2019) suggested that related items (descendants from a common ancestor in an ultrametric tree of items) may be efficiently represented in a sparse and condensed manner by representing their common “ancestor” or generator plus differences of each item from it. Thus, representations of set and category items might inherently include representation of mean and prototype, respectively. Prototype theory is not new, of course, but it is strengthened by the current finding of the resemblance of categorization with set perception.

There is some debate concerning the relationship between object familiarity and category typicality (Nosofsky, 1988; Palmeri & Gauthier, 2004; Shen & Reingold, 2001). Responses are more rapid for familiar objects (Wang, Cavanagh & Green, 1994; familiar faces: Ramon, Caharelô, & Rossion, 2011; familiar words: Glass, Cox & LeVine, 1974; familiar size: Konkle & Oliva, 2012) or typical objects (Ashby & Maddox, 1991, 1994; McCloskey & Glucksberg, 1979; Rips et al., 1973; Rosch, 1973; Rosch, Simpson, & Miller, 1976), but familiar objects are often deemed more typical (Iordan, Green, Beck, & Fei-Fei, 2016; Malt & Smith, 1982) and unfamiliar objects are quickly rejected from category membership (Casey, 1992). Thus, our use of reaction times for judging typicality may have included familiarity, and our finding that participants chose more typical objects may have included choice of more familiar objects. Nevertheless, while Rosch (1973) found that categorization responses are faster to prototypical objects, Ashby, Boynton, and Lee (1994) did not find a “meaningful correlation between response time and stimulus familiarity” when not related to category. In our experiments, choosing the prototype or familiar object as having been SEEN when it was not shown in the trial sequence, is surprising and not expected based only on familiarity. Rather, such a result would be consistent with a situation where sequence object representations included a representation of their prototype (e.g., see Benna & Fusi, 2019).

Our results resemble the Deese–Roediger–McDermott (DRM; Roediger & McDermott, 1995) finding that when presented with a list of related words, participants recall a nonpresented “lure” word with the same frequency as the presented words. In the DRM paradigm, participants study lists of words (e.g., tired, bed, awake, rest, dream, night, blanket, doze, slumber, snore, pillow, peace, yawn, drowsy) that are related to a nonpresented lure word (e.g., sleep). On a later test, participants often claim that they previously studied the related lure words. Similarly, it was found that after learning a set of distortions of a random dot pattern, participants learn the undistorted pattern—the prototype—more easily than a new distortion, though only after a first viewing (Posner & Keele, 1968). These results may be added to the ensemble and categorization results, relating different situations—semantic and perceptual—where perceiving related items induces representation and recall of the mean or prototype processes, suggesting that similar processes may underlie them.

Such recall is referred to as “false” memories, since false recognition of the related lure words is indistinguishable from true recognition of studied words (Gallo 2006; Schacter & Addis, 2007). Our results, too, reflect “false” memories, since participants indicate recall of items that were not presented in the sequence. This is equally true for our study of category prototype recall and our studies, and those of many others, of set ensemble presentation and recall of the set mean—even in its absence from the presented sequence. Nevertheless, the term “false memory” is generally used in reference to recall of events (Zaragoza, Hyman, & Chrobak, 2019) and narratives (Frenda, Nichols, & Loftus, 2011) that did not occur or were not narrated. Finding false memory of category prototype certainly extends this notion from abstract mean parameter (size, orientation, brightness, etc.) to more concrete objects and semantic categories, but this is still far from false episodic memory. Further study is required to decide if these different types of false memory are related, and if so, what is the relationship between them.

Perceiving category exemplars in terms of the category prototype may be the source of categorical priming (e.g., Fazio, Williams, & Powell, 2000; Ray, 2008), whereby responses to unseen exemplars (and in particular to the category prototype) are faster when primed by previously perceiving another category exemplar. Interestingly, similar effects have been found for sets (Marchant & de Fockert, 2009), and there is even negative priming for unconscious viewing of single unusual shapes (DeSchepper & Treisman, 1996).

## Conclusions

We conclude that while observing the projected images, participants first, implicitly generalized them into a category. Then, at the membership test, they use this categorical context to classify the probability of presence within the sequence of the test images. That is, when visual memory capacity is insufficient, then this implicit categorical context affects their judgment. If indeed categorizations are executed by similar computations as in statistical perception of the visual system, then it is possible that these are only particular embodiments of a general system, which efficiently determines our perception and behavior. It is especially poignant that set mean perception and categorization, which help behaving in a too-rich and too-complex environment by applying shortcuts to perception, may share perceptual-computational mechanisms, perhaps at different cortical levels. We have suggested that the neural mechanism used is a population code (Georgopoulos et al. 1986) that encodes both the mean and the range of the stimulus set (Hochstein, 2016a, 2016b; Pavlovskaya, Soroker, Bonneh, & Hochstein, 2017a, 2017b; see also Brezis, Bronfman, Jacoby, Lavidor, & Usher, 2016; Brezis, Bronfman, & Usher, 2018). Using a population code to determine set mean answers the question of how the visual system computes mean values without knowing values for each element separately (whether represented when viewed and forgotten, or never explicitly represented). Due to broad tuning and overlap of neuron receptive fields, a population code is necessarily used for perceiving individual element values and may be used directly, with a broader range of neurons over space and time, to perceive set mean values. We now suggest the same type of population code may be used for categorization. Category prototype and boundaries could be the read out of fuzzy representations of multiple exemplars. It has already been suggested that ensemble summary statistics might serve as the basis for rapid visual categorizations (Utochkin 2015).

A distinction was made between automatic, intuitive global-attention scene gist perception, using vision at a glance, versus explicit, focused-attention vision with scrutiny (Hochstein & Ahissar, 2002). Gist is acquired automatically and implicitly by bottom-up processing, and details are added to explicit perception by further top-down guided processes. The current study demonstrates that even when it is observers’ intention to detect and remember the details of each image in a sequence—an intention that in this case often leads to failure—nevertheless, the automatic, implicit process of gist perception succeeds in acquiring both set and category information.

A question that still needs to be addressed is the cerebral correlates of mechanisms underlying these processes. An investigation using physiological techniques (fMRI or EEG), while participants perform behavioral tasks, as in the current study, might indicate brain regions or electrophysiological patterns of activity that are specific to systems that generate these automatic representations. Such a study might also test the notion that similar sites perform set mean and range perception as well as categorization.
